# Transcriptome profiling of *Toona ciliata* young stems in response to *Hypsipyla robusta* Moore

**DOI:** 10.3389/fpls.2022.950945

**Published:** 2022-08-25

**Authors:** Huiyun Song, Yue Li, Zhi Wang, Zhihao Duan, Yueyang Wang, Endian Yang, Qingmin Que, Xiaoyang Chen, Pei Li

**Affiliations:** ^1^College of Forestry and Landscape Architecture, South China Agricultural University, Guangzhou, China; ^2^Guangdong Key Laboratory for Innovative Development and Utilization of Forest Plant Germplasm, South China Agricultural University, Guangzhou, China; ^3^State Key Laboratory for Conservation and Utilization of Subtropical Agro-Bioresources, South China Agricultural University, Guangzhou, China

**Keywords:** RNA-seq, *Toona ciliata*, *Hypsipyla robusta* Moore, terpene synthase, jasmonic acid, transcription factor

## Abstract

*Toona ciliata* is a traditional woody plant that can be used as a medicinal material in China. The extracts of its roots, stems, leaves, and flowers all have a wide range of bioactive compounds. However, *T. ciliata* has been facing an unresolved pest problem caused by *Hypsipyla robusta* Moore (HRM), which seriously affects its growth and development. In this study, the expression level of *TcMYB3* gene reached the maximum (28-fold) at 12 h and transcriptome sequencing of young stems eaten by HRM for 0, 3, 12, and 21 h were performed. A large number of differentially expressed genes (DEGs) were identified including jointly up-regulated genes (263) and down-regulated genes (378). JA synthesis and signaling transduction, terpene biosynthesis, and MAPKs signaling pathway were analyzed in depth and found that *TcOPR3, TcJAR1, TcJAZs*, and *TcTPS9* genes possessed anti-insect potential. Moreover, MYB and ERF transcription factor (TF) families were significantly strengthened to the point that they may participate in induced defense mechanisms in *T. ciliata*. These data not only provide insights into the molecular mechanisms in resistance of *T. ciliata* to HRM but also helps to explore the new biocontrol strategies against insects in eco-friendly woody plants.

## Introduction

*Toona ciliata* is a wild and endangered plant under the national level II key protection, which belongs to Meliaceae family (Li et al., 2018). It is also called “Chinese Mahogany” because its wood has a beautiful texture, straight dry shape, russet hues, and is soft and corrosion-resistant (Song et al., 2020b). It is often used as a high-end furniture material for export and has high economic value (Song et al., 2020a). The extracts from leaves, stems, roots, and flowers of *T. ciliata* have antibacterial, anti-tumor, anti-glycation, and cytotoxic functions, which were used as medicine to treat gastric ulcers (Malairajan et al., 2007; Zhu et al., 2019; Shi et al., 2020). However, the *Hypsipyla robusta* Moore (HRM) adult worms prefer to lay eggs on the *T. ciliata* leaves, so the stems and apical buds are susceptible to damage from *H. robusta* larvae feeding on them. This damage will cause a lot of side branches to grow, eventually forming a “multi-headed tree” (Newton et al., 1993). Repeated attacks by HRM greatly reduce the quantity and value of wood. Pests of *T. ciliata* are not only a regional problem, but also a global problem as observed by studying the main areas of *T. ciliata* overseas, such as Australia and Brazil, which are also threatened by *H. robusta* (Cunningham and Floyd, 2004). Considerable research has been conducted on *H. robusta*, and many prevention methods have been proposed, but none of them have achieved success commercially (Abraham et al., 2014). Chemical applications are considered impractical in terms of environment and economy because of the inaccessibility of larvae, heavy rains, and high temperatures in tropical areas (Nuraeni and Nuroniah, 2020). The number of natural enemies is very small, indicating that the natural population cannot achieve the required level of biological control (Newton et al., 1993).

Oxophytodienoate reductase 3 (OPR3) is a key enzyme in the JA synthesis pathway. The 12-oxo-phytodienoic acid (OPDA) is transferred to peroxisome and reduced by OPR3. In tomatoes, OPR3 was silenced by RNA interference (RNAi), and it was found that OPR3-RNAi plants contained wild-type levels of OPDA but failed to accumulate JA or JA-Ile after wounding. Meanwhile, the loss of these JA/JA-Ile -dependent defense traits rendered them more attractive to the specialist herbivore *Manduca sexta* with respect to feeding and oviposition (Bosch et al., 2014). JA is transported to the cytoplasm to form the active form of jasmonic acid-isoleucine (JA-Ile) under the catalysis of jasmonoyl amino acid conjugate synthase (JAR1; Wasternack and Song, 2017). The jasmonate ZIM domain (JAZ) is a zinc finger protein, including Jas and ZIM domain, which plays the role of “suppressor” in the JA pathway (Ju et al., 2019). In the absence of stress, the JAZ protein interacts with novel interactors of JAZ (NINJA) to recruit the inhibitor topless (TPL), which makes JAZ proteins to inhibit the transcriptional activation of the JA response gene by interacting with MYC2 (Howe and Yoshida, 2019). When JA accumulation, JA-Ile is formed and transported to the nucleus that facilitates the interaction of JAZ with coronatine insensitive1 (COI1). Moreover, COI1 is the F-box component of E3 ubiquitin ligase SCF^COI1^ required for all JA-dependent responses tested so far (Thireault et al., 2015). Ubiquitination of the JAZ protein would lead to its proteasomal degradation and release TFs to modulate the expression of JA-responsive genes, thereby regulating defenses and growth (Thireault et al., 2015). In cotton, GhJAZ2 interact with GhbHLH171 and inhibit its transcriptional activity, and negative feedback regulation of the JA-mediated defense response (He et al., 2018).

The MVA pathway is regulated by six catalytic enzymes, namely, acetyl-CoA acetyltransferase (*AACT*) gene, hydroxymethylglutaryl-CoA synthase (*HMGS*) gene, Hydroxymethyl glutaryl-CoA reductase (*HMGR*) gene, phosphomevalonate kinase (*PMK*) gene, mevalonate diphosphate decarboxylase (*MDC*) gene and mevalonate kinase (*MK*) gene (Vranová et al., 2013; Yue et al., 2015). Six genes on the MEP pathway have been identified, namely, five 1-deoxy-D-xylulose 5-phosphate synthase (DXS), 1-Deoxy-D-xylulose 5-phosphate reductoisomerase (DXR), two 2-C-methyl-D-erythritol 4-phosphate cytidylyltransferase (MCT), 4-(cytidine 5-diphospho)-2-C-methyl-D-erythritol kinase (CMK), three 4-hydroxy-3-methylbut-2-enyl-diphosphate synthase (HDS), and three 4-hydroxy-3-methylbut-2-enyl diphosphate reductase (HDR). Terpenoids are synthesized by terpenoid synthases on MEP and MVA. Terpene synthases (TPSs) are a diverse class of enzymes which catalyzes the biosynthesis of hemiterpenes (C5), monoterpenes (C10), sesquiterpenes (C15), or diterpenes (C20) using the substrates DMAPP, GPP, FPP, or GGPP, respectively (Abbas et al., 2017). Studies have shown that terpenoids have anti-insect effects. For example, in maize, *TPS10* can produced the sesquiterpene that can provide a volatile signal for the indirect defense of the plant against herbivore attack (Schnee et al., 2006).

When suffering from pests, plants will activate a series of effective defense mechanisms (Nalam et al., 2019). For example, the physical and chemical barriers and defensive substances (its existence does not depend on the environment) against insects are called constitutive defenses (Fürstenberg-Hägg et al., 2013). In addition, after plants are invaded by insect pests, a large number of defensive compounds are synthesized to activate defense related genes expression to produce self-defense response, which is similar to immune response and called inductive defense (Wang et al., 2020). For example, when maize is harmed by insects, its immune response will be activated. Then it will release single maize sesquiterpene to attract natural enemies (Schnee et al., 2006). Studies have shown that by releasing volatile substances, such as terpenoids, aromatic compounds and green leaf volatiles, plants attract predatory or parasitic natural enemies of insect pests that play an indirect defense role (Clavijo McCormick et al., 2014). Inducible defense will also cause the body to produce a series of complex signal networks triggered by hormones and so on (Mangwanda et al., 2015). Jasmonic acid (JA), salicylic acid (SA), and ethylene (ET) are important barriers in plant-induced defense responses. The three pathways' signals are transmitted to the nucleus and initiate a series of defense responses. The JA pathway is induced by the insects' chewing mouthparts during feeding and mechanical damage, and the ET signaling pathway works in concert with JA. The SA pathway is mainly induced by insect feeding with piercing and sucking mouthparts. At the same time, abscisic acid (ABA), auxins, and cytokinins (CKs) also play important regulatory roles (Thaler et al., 2012; Papadopoulou and Van Dam, 2017). Each signal pathway has a synergistic or resistance effect, providing a mechanism for plants and the defense mechanism of layer plasticity (Nguyen et al., 2016). When the brown planthopper (*Nilaparvata lugens*) attacked rice, the JA and ET pathways was induced to coordinate and negatively regulate defenses (Ma et al., 2020). Therefore, cultivating insect-resistant species of *T. ciliata* by means of gene editing has become an effective means to completely solve the problem.

Our team found that there is no insect-resistant *T. ciliata* provenance in nature, so it's important to have a deep dig and understanding of the induced defense mechanism. RNA-Sequencing (RNA-seq) is a new generation of high-throughput sequencing technology developed in recent years (Oates et al., 2015). Through high-throughput sequencing, almost all transcript sequence information and expression level of a specific cell or tissue in a certain state can be obtained comprehensively and quickly (Ayturk, 2019). RNA-seq has the advantages of high throughput, high resolution, low cost, high accuracy, and is applicable to any species (Słomnicka et al., 2021). It has been widely used to explore the defense mechanisms of plants against pests and has made great progress. In *Eucalyptus*, 698 and 1,115 significantly differentially expressed genes from the resistant and susceptible interactions, respectively, were found. In addition, terpenoids profiles were significantly different under *Leptocybe invasa* stress by comparing the transcriptome (Oates et al., 2015). The main defense pathway of corn against Asian corn borer is JA defense signaling pathway (Yang et al., 2015). By analyzing the transcriptome of an apple (*Malus ieversii*) under *Agrilus mali* stress, different expressed genes were mainly involved in the signal transduction pathway of plant hormones and in the synthesis of compounds such as terpenes, quinones, flavonoids, and JA (Mei et al., 2020). Meanwhile, Giovino et al. (2015) studying the transcriptome of *Phoenix canariensis* activated by feeding by *Rhynchophorus ferrugineus* and found JA and SA pathways were induced to participate in the defense against insects. The Illumina HiSeq 2000 sequencing platform was used to sequence the transcripts of cotton buds fed by *Anthonomus grandis* larvae and a total of 443 differentially expressed genes were identified, and 432 were *Arabidopsis* orthologous genes, including ET and JA signal genes (Artico et al., 2014). However, as far as we know, the response mechanism on transcriptional level of *T. ciliata* against *H. robusta* has not yet been reported.

In this study, RNA-seq was performed on the tender stems of *T. ciliata* that were gnawed at 0, 3, 12, and 21 h by *H. robusta*. We then sorted out the relevant genes and pathways of endogenous insect resistance to clarify the molecular mechanism in response to *H. robusta*. Currently, the organic combination of conventional breeding and plant molecular biology technology is an important method for the genetic improvement of *T. ciliata*. This study provides an insect-resistant gene library for breeding of insect-resistant varieties, which is also important for the prevention and control of *H. robusta*.

## Materials and methods

### Plant materials and treatment

**Figure 1:**
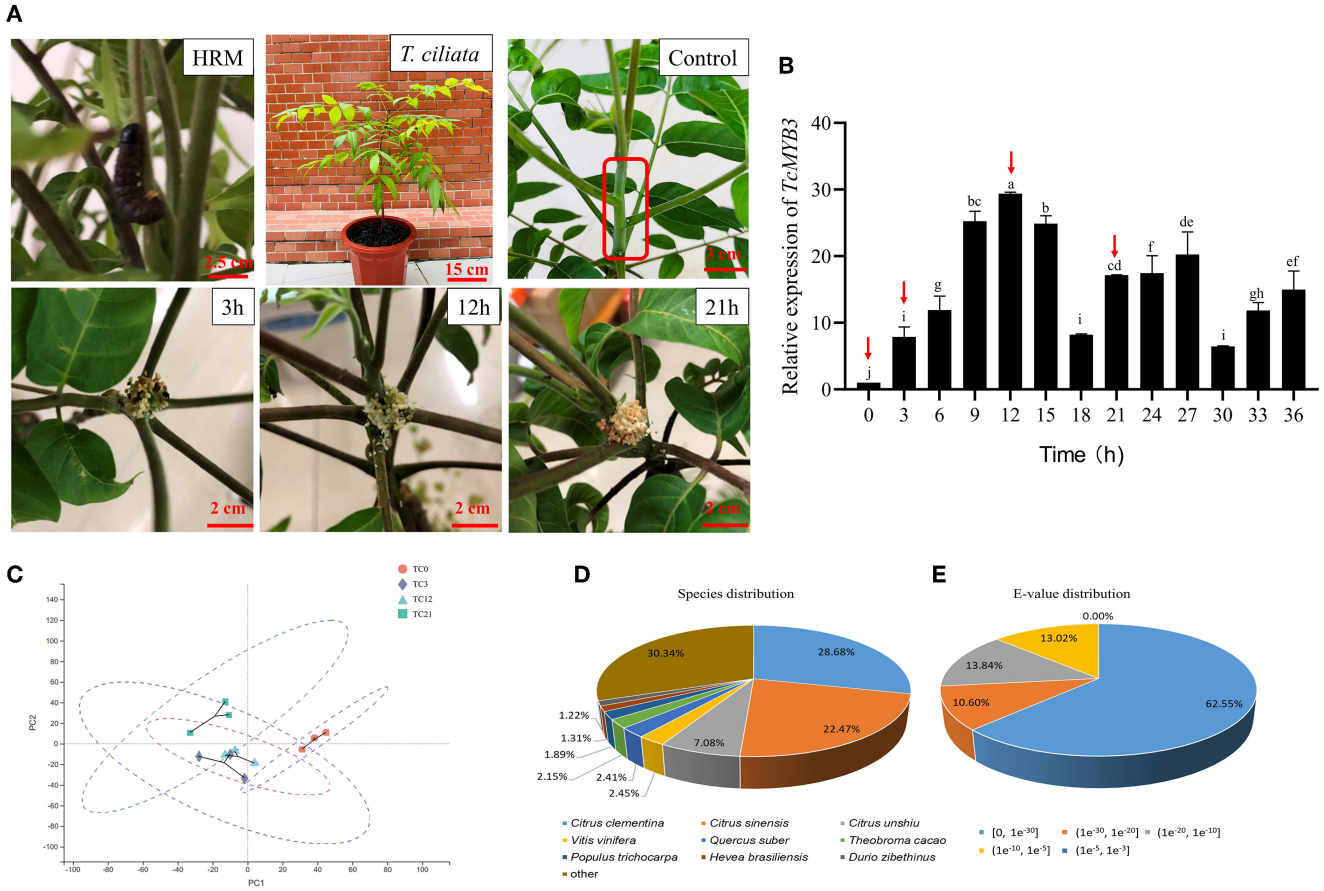
**(A)** The picture of *HRM and T. ciliata* was fed 3, 12, and 21 h by HRM. The young stem in the red box is control. **(B)** The relative expression of *TcMYB3* in different times of HRM feeding. **(C)** PCA analysis of transcriptome among four samples. The English letters on the histogram are calculated according to Duncan's multiple range test, and different letters represent significant differences between the different groups at *P* ≤ 0.05. **(D)** Species distribution of the top 10 and **(E)** *E*-value after a single gene BLAST. The cut-off values for BLAST search set at 1.0e^−5^.

The experiment materials were healthy 3-year-old clones of *T. ciliata*, which grew in the College of Forestry and Landscape Architecture in South China Agricultural University under natural light (SCAU, Guangzhou). *T. ciliata* young stems are endangered by the 1st and 2nd instar larvae of HRM, which are difficult to cultivate in the laboratory. Therefore, the HRM larvae used in the experiment was collected from the young tenders that had been eaten by the insects in Qilin North Experimental Base (SCAU) and no additional feeding of HRM was required. On the contrary, collected HRM were starved in a blank culture dish for 12 h. In addition, in order to make HRM consistent, the second instar larvae of HRM with a body length of 4–7 cm were selected. Sufficient numbers of HRM larvae were collected to ensure that each HRM only gnawed on one *T. ciliata*. Then HRM were placed on the young stem of *T. cilata* (3–4 cm away from the terminal bud) and each *T. ciliata* was inoculated with one HRM. When the HRM began to bite *T. ciliata*, the time was counted. The young stems bitten by HRM for 0, 3, 6, 9, 12, 15, 18, 21, 24, 27, 30, 33, and 36 h were taken respectively, and each time point had three repetitions (Figure 1A). Since the body of HRM had already entered the young stems after 3 h, which resulted in the whole young stems being eaten, the whole tender stems were collected and quickly frozen in liquid nitrogen and stored at −80°C.

### Identification samples of RNA sequencing

When plants are subjected to external stress, a series of dynamic changes of endogenous genes will occur. Previous studies have shown that MYB transcription factor (TFs) not only confer resistance to the brown planthopper by regulating the phenylalanine ammonia lyase pathway in rice (He et al., 2020b), but also synergistically regulate the defense of phloem against English grain aphids in wheat (Zhai et al., 2017). Therefore, the relative expression level of *TcMYB3* in *T. ciliata* under HRM feeding was detected to preliminarily determine the expression pattern of the endogenous gene in response to HRM feeding. *HIS1* was selected as the reference gene (Song et al., 2020a). The samples for RNA sequencing were determined though the relative expression level of *TcMYB3*. The first sampling point was the time when the relative expression of *TcMYB3* increased for the first time, the second sampling point was the time when the relative expression of *TcMYB3* increased to the maximum, and the third sampling point is equal to the second sampling point plus the difference between the second and first sampling points. Meanwhile, untouched trees were the control group. RNA sequencing was performed on samples of untouched trees and the three sampling points.

### RNA extraction, library preparation, and sequencing

Total RNA of *T. ciliata* young stem was extracted by TRIzol^®^ Reagent (Plant RNA Purification Reagent for plant tissue, Invitrogen), then genomic DNA was eliminated through DNase I (TaKara). Total RNA quality was determined by a 2100 Bioanalyser and quantified using the ND-2000. Finally, the high-quality RNA sample (OD260/280 = 1.8–2.2, OD260/230 ≥ 2.0, RIN ≥ 8.0, 28S:18S ≥ 1.0, >1 μg) was used to construct the sequencing library (Jung et al., 2018). Library construction, and sequencing were performed at Shanghai Majorbio Bio-pharm Biotechnology Co., Ltd. according to the manufacturer's instructions (Illumina, San Diego, CA). The transcriptome libraries of *T. ciliata* young stems were prepared using Illumina TruSeqTM RNA sample preparation Kit (San Diego, CA). First, poly-A tail mRNA was enriched from 5 μg total RNA with oligo (DT) magnetic beads. Then the fragmentation buffer was added to randomly break the mRNA into small fragments of about 200 bp (Jung et al., 2018). Then, a single strand cDNA was synthesized by using a superscript double stranded cDNA asynchronous (Invitrogen, CA) kit and adding six base random primers (Illumina) with mRNA as inversion, and then two strand synthesis was carried out to form a stable double strand structure (Jung et al., 2018). Double stranded cDNA is a sticky end. End repair mix is added to make it a flat end, and then a base is added at the 3' end to connect the Y-shaped connector. After PCR enrichment, 200–300 bp bands were recovered with 2% agarose gel. After quantification by tbs380 (PicoGreen), the library used Illumina Hiseq X Ten / NovaSeq 6000 sequencing platform for high-throughput sequencing, and the sequencing reading length was PE150 (Jung et al., 2018).

### Differential expression analysis and functional enrichment

The original paired end reading was determined by SeqPrep and Sickle with default parameters for trimming and quality control. Then clean data from samples (young stems of *T. ciliata*) were used to do *de novo* assembly with Trinity (Grabherr et al., 2011). BLASTX was used to search all assembled transcripts against NCBI protein non-redundant (NR), COG, and Kyoto Encyclopedia of Genes and Genomes (KEGG) databases to identify the protein with the highest sequence similarity to a given transcript to retrieve its functional annotation, and typical cut-off *E*-values <1.0 × 10^−5^ were set. The BLAST2GO (Conesa et al., 2005) program was used to obtain GO annotations of uniquely assembled transcripts to describe biological processes, molecular functions, and cellular components. Metabolic pathway was analyzed by KEGG (Kanehisa and Goto, 2000).

### Screening and identification of insect resistance genes

Plant hormone biosynthesis and signal transduction pathways play a vital role in the defense process induced by insect feeding. Three types of plant volatile compounds can be induced, including terpenes, fatty acid derivatives, and phenyl/phenylpropanes (derivatives of shikimic acid), which have important anti-insect functions. Therefore, this study combined the results of differential gene function annotation and the KEGG pathway to analyze the terpene synthesis and JA biosynthesis and signal transduction pathways. The synthetic transduction schematic diagrams of the two pathways are drawn through the reference, and the heat maps of related gene expression are drawn by TBtools (Chen et al., 2020). NCBI-Blast queried the homologous sequence to initially confirm the gene family, then it was processed by NCBI-Batch-CDD through predicting the gene functional domain for the second confirmation. Meanwhile, Target P 1.1 Server (https://services.healthtech.dtu.dk/service.php?TargetP-2.0) and WoLF PSORT (https://wolfpsort.hgc.jp/) were combined to predict protein subcellular location.

### Selection of key genes and phylogenetic analysis

In the RNA-seq database, genes with more than 10-fold up-regulated gene expressions were regarded as key genes. The protein sequences of the key genes family were from RNA-seq library because there is no genome-wide database of *T. ciliata* when performing transcriptome sequencing. In order to gain insight into the evolutionary relationship of the key gene's family, homologous sequences are distinguished by constructing phylogenetic trees by MEGA7. The trees were constructed with the Maximum Likelihood method (ML) with 500 bootstraps. Meanwhile, the protein sequences of AtLOXs, AtJAZs, AtDXSs, CsDXSs, OsDXSs, PtrDXSs, and AtTPSs were downloaded from the Phytozome database.

### Analysis of transcription factors

By analyzing the domain information contained in the transcription product, the TF's prediction and family analysis of the transcript was carried out. Then the HMMER analysis method was used to compare with the database PlantTFDB (http://planttfdb.cbi.pku.edu.cn/) to obtain the same homologous TFs information. Finally, the specific information of transcription factor analysis and transcription factor family were counted.

### Quantitative real-time PCR (Q-PCR)

According to CDS sequence from RNA-seq database, Q-PCR primers were designed by NCBI-PrimerBlast (https://www.ncbi.nlm.nih.gov/tools/primer-blast/; Supplementary Table 1, Supplementary Figure 1). The template cDNA of Q-PCR includes the tender stems and leaves under 0, 3, 12, and 21 h HRM feeding. *HIS1* were selected as reference genes. The Q-PCR reaction mixture consisted of 10 μL ChamQ Universal SYBR qPCR Master Mix (Vazyme), cDNA 2 μL, each primer 0.4 μL (10 μM), 7.2 μL ddH_2_O. Q-PCR reaction was performed in LightCycler480 (Roche Molecular Biochemicals, Mannheim, Germany) with an optical 96-well plate. The reaction procedure is 95°C for 30 s; 95°C for 15 s, 60°C for 20 s, 72°C for 10 s, 40 cycles; performing melting curve analysis at 65–95°C, and then through the peak diagram of the melting curve to determine the specificity of primers. There are three biological replicates for each sample and three technical replicates for each biological replicate. According to the Ct value obtained in the experiment, the 2^−ΔΔCt^ method was used to calculate the expression under different conditions. Meanwhile, Duncan's multiple test analysis of Q-PCR data by R software and linear regression analysis with GraphPad Prism 8.

### Assessment of JAs

The quantification of plant hormones was performed as previously described with slight modifications (Dobrev and Vankova, 2012). The young stems eaten by HRM for 0, 3, 12, and 21 h were ground into powder with a grinder (30 Hz, 1 min). First, 50 mg of the ground sample was weighed, and an appropriate amount of internal standard was added. Extraction was then performed with 1 mL of methanol methanol/water/formic acid (15:4:1, v/v/v). The extract was concentrated and reconstituted with 100 μL of 80% methanol/water solution, passed through a 0.22 μm filter, and placed in a sample vial for ultra-performance liquid chromatography-mass spectrometry (LC-MS/MS), as described previously by Yamamoto et al. (2015).

## Results

### A preliminary study on the response pattern of *T. ciliata* to HRM feeding

The response of plants to insect pests will change dynamically with time. Therefore, Q-PCR was used to quantify the expression of *TcMYB3* at different times of HRM feeding and gene pattern of *T. ciliata* were initially understood in response to HRM feeding (Figure 1B). The results showed that the relative expression level of *TcMYB3* gradually increased from 3 to12 h, reaching the maximum value at 12 h. And it was in a declining-increasing-declining-increasing fluctuation state at 12–36 h, but the relative expression levels were all lower than 12 h. Therefore, control (TC0, untouched trees as control), 3 h (initial response stage, TC3), 12 h (strongest response stage, TC12), and 21 h (response decline stage and followed the time rhythm, TC21) samples were selected for RNA-seq sequencing, to further explore the response pattern of *T. ciliata* against HRM.

### RNA-seq sequencing, assembly, and annotation

The young stems of *T. ciliata* fed on by HRM for 0, 3, 12, and 21 h were selected as RNA-seq sequencing materials (Figure 1A). The quality evaluation of RNA-seq results show that the 12 samples have 7.82G, 7.28G, 7.50G, 7.91G, 8.01G, 6.99G, 7.89G, 7.47G, 6.62G, 6.51G, 7.65G, and 6.67G clean bases. Q20 is >98%, Q30 is >94%, and error rate is <0.025%, which indicates that the quality of sequencing data is reliable (Supplementary Table 2). Principal component analysis (PCA) was performed among the four groups of samples on gene expression and results showed no abnormal values (Figure 1C). However, perhaps due to the short time interval between TC3 and TC12 samples, there is not a large difference (Figure 1C), but it is acceptable for subsequent analysis. A review of the transcriptome database of *T. ciliata* young stems fed on by HRM shows as Supplementary Table 3. About 84,227 unigenes and 76.19 million bases were generated. N50 length (1,616 bp), TransRate score (0.14238) and BUSCO score (72%) illustrate the integrity of the transcriptome assembly is very good. In addition, the length of 42,459 unigenes are shorter than 500 bp and 1,060 unigenes are longer than 4,500 bp (Supplementary Figure 2A). Then, all unigenes sequences were aligned with six public databases and results show that 41,568 unigenes obtained annotation information, accounting for 49.35% of all unigenes (Supplementary Table 4). In Nr database, the top three species most similar to *T. ciliata* belong to *Citrus* (Figure 1D), which suggest *T. ciliata* is similar to *Toona sinensis* but was closer to the genus *Citrus* (Ji et al., 2021). In addition, based on BLASTX search against the NR database with an *E*-value cut-off of 1.0e^−5^, 39,370 unigenes returned a significant BLAST hit (Supplementary Table 4). Among them, 62.55% annotated unigenes had very strong homology with the top matching sequence (0 < *E*-vale < 1e^−30^), 10.60% shows strong homology (1e^−30^ < *E*-vale < 1e^−20^) and the homology of the remaining unigenes is weak (1e^−20^ < *E*-vale < 1e^−3^; Figure 1E).

### Expression analysis of unigenes gene

In RNA-seq analysis, the expression level of genes is estimated by counting the number of reads located in the genomic region or gene exon region (Yue et al., 2015). Transcripts per million (TPM) was introduced to facilitate comparisons between samples. TPM stands for transcripts per million, the sum of all TPM values is the same in all samples, so TPM values represent relative expression levels, which should in principle be comparable between samples. The homogenization process of TPM makes the total expression level in different samples consistent, so that the expression level can be compared more intuitively (Zhao et al., 2020). The distribution of unigene expression level in 12 samples of *T. ciliata* is shown in Supplementary Figure 2B, the most concentrated area of each sample is when log_10_TPM = −0.5. The number of unigenes with log_10_TPM > 2 and log_10_TPM < −1 is relatively small, showing polarization. The unigenes expression level are divided into five expression levels according to the TPM interval, which includes 0–1, 1–3, 3–15, 15–60, and >60 (high expression level) (Supplementary Table 5). The number of genes of TC21, TC12, and TC3 TPM value >60 is larger than that of TC0, indicating that there are more genes in *T. ciliata* that are induced to express against the HRM (Supplementary Table 5).

### DEGs analysis under HRM feeding

**Figure 2:**
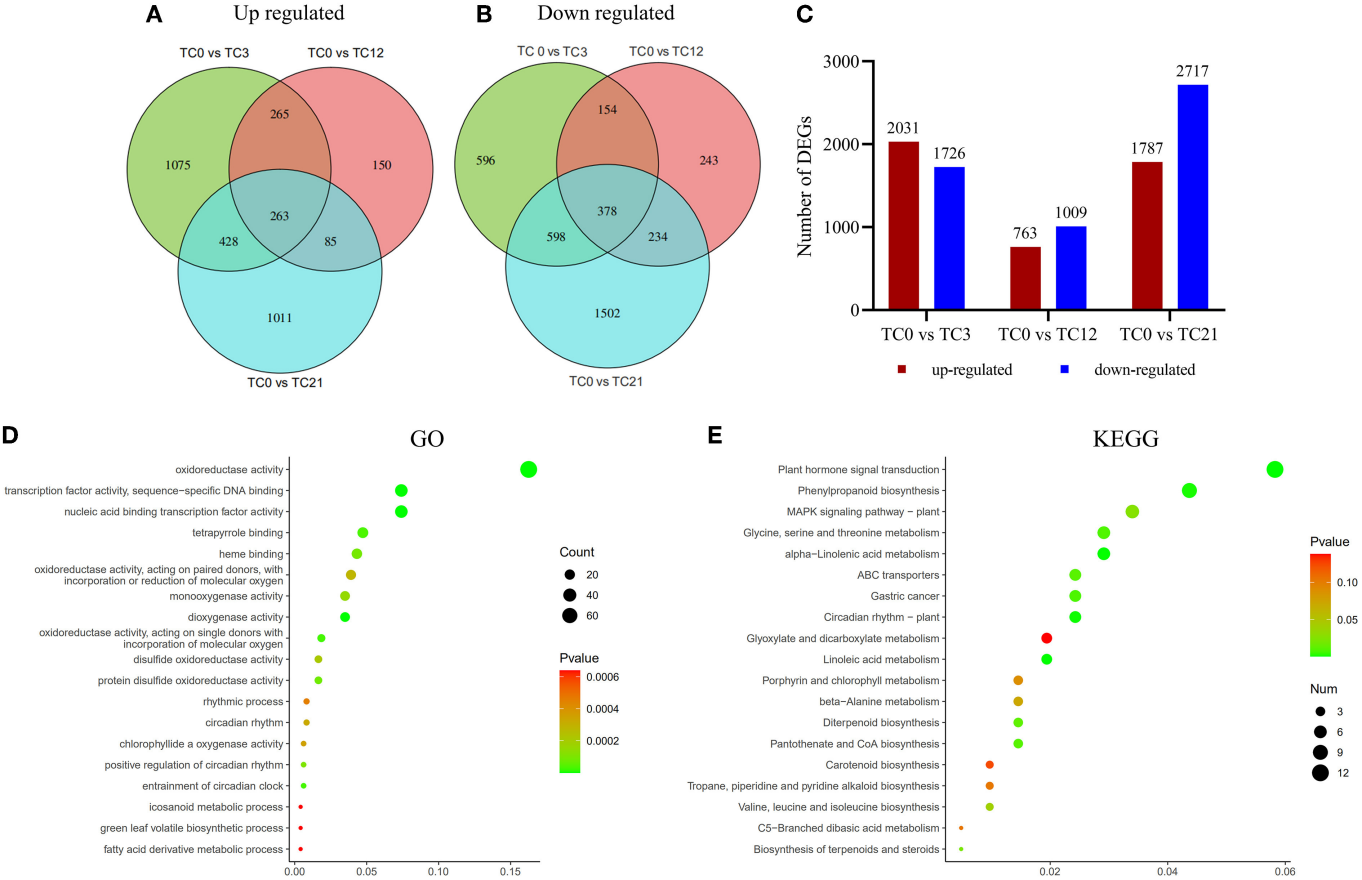
Gene expression comparisons. **(A)** Venn diagram of number of up regulated DEGs. **(B)** Venn diagram of number of down regulated DEGs. **(C)** Changes in gene expression profile. The numbers of up-regulated and down-regulated genes between TC0 and TC3, TC0 and TC12, TC0 and TC21 are summarized. Results from the GO **(D)** and KEGG **(E)** pathway enrichment analysis for DEGs. Color indicates the *P*-value. The 19 most enriched pathways are listed (left).

In order to identify significant DEGs of *T. ciliata* in different time periods under HRM feeding. First, TC3, TC12, and TC21 were compared with TC0 to obtain DEGs (up regulated and down regulated) of TC0 vs. TC3, TC0 vs. TC12, and TC0 vs. TC21, respectively. Then, in order to distinguish the DEGs that were jointly up-regulated or jointly down-regulated, the up-regulation or down-regulation of TC0 vs. TC3, TC0 vs. TC12, and TC0 vs. TC21 were compared. The conditions for screening DEGs are |log_2_(foldchange)| > 1 and *p*-adjust <0.05. The results show that there are 263 and 378 genes that are jointly up-regulated and down-regulated, respectively (Figures 2A,B). After 3 h of stress, the number of genes that are up-regulated (2,031) and down-regulated (1,726) are higher than the number of genes that are up-regulated (763) and down-regulated (1,009) after 12 h of stress. And the number of up-regulated (1,787) and down-regulated (2,717) genes rise to the maximum after 21 h of stress (Figure 2C). It shows that different and more complex biological events occurred in *T. ciliata* at 3 and 21 h.

For exploring the biological changes of *T. ciliata* during the HRM feeding, all DEGs were analyzed by GO enrichment (Figure 2D). Most DEGs are mainly enriched in oxidoreductase activity, dioxygenase activity, protein disulfide oxidoreductase activity, etc. In addition, all DEGs were subjected to KEGG enrichment analysis (Figure 2E). Among them, the five most enriched metabolic pathways are plant hormone signal transduction, phenylpropanoid biosynthesis, MAPK signing pathway, Glycine, serine, and threonine metabolism, and α-linolenic acid metabolism.

### Analysis of putative insect-resistant genes in JA pathway

JA is an important signal in many developmental processes such as seed germination, root and whole plant growth, stamen development, and senescence (Wasternack and Song, 2017) and JA regulates the induction of direct and indirect defenses against herbivores. More importantly, α-linolenic acid metabolism and plant hormone signal transduction pathways closely related to JA are significantly enriched under the HRM attack in *T. ciliata*. Therefore, the genes in JA biosynthesis and signal transduction pathways were excavated and analyzed.

**Figure 3:**
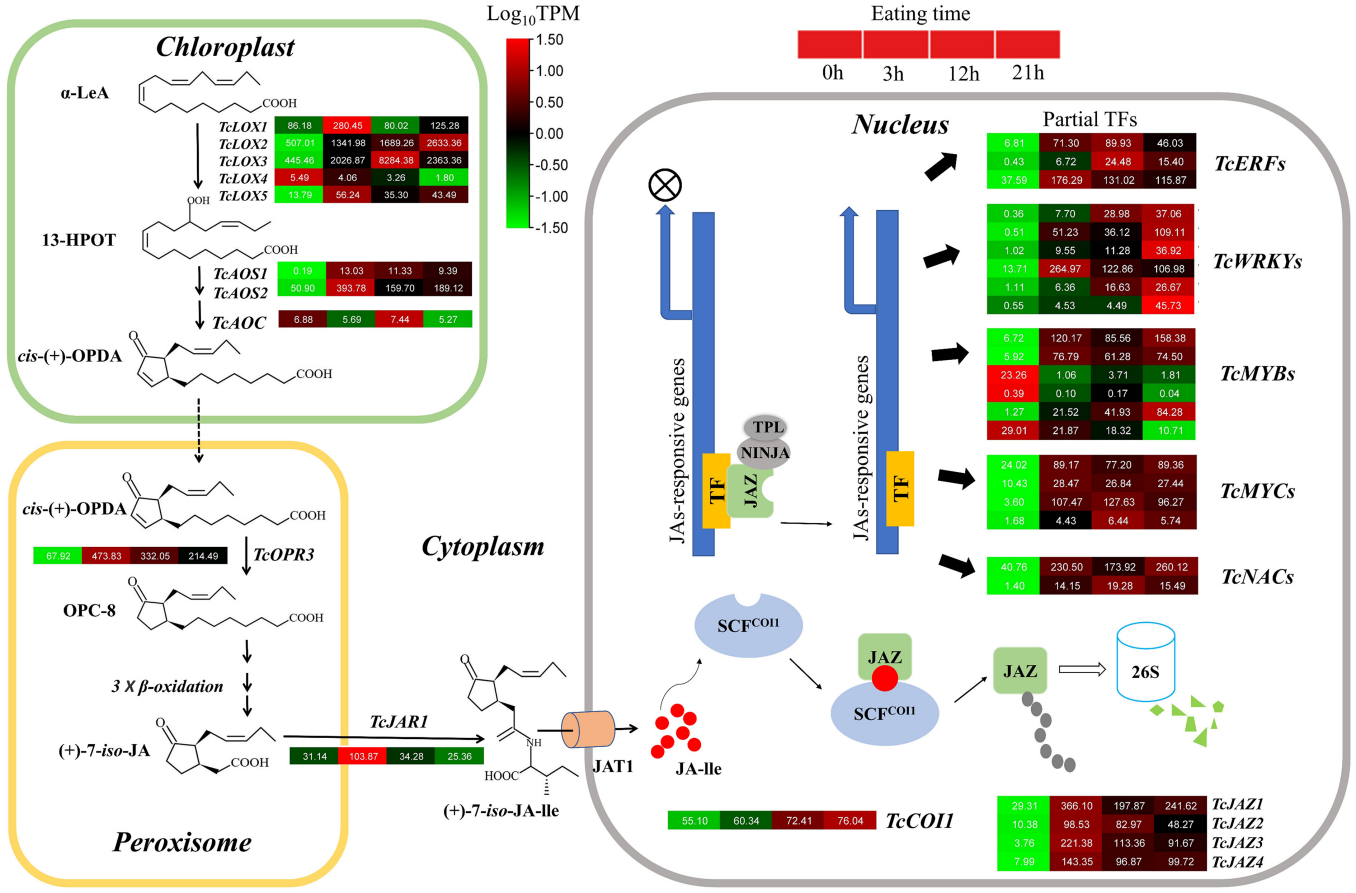
Gene expression pattern diagram of JA metabolism and signal transduction pathway. The names of enzymes in each catalytic stage are represented by abbreviations and italics in bold. Color scales indicate gene expression levels (Log_10_TPM) at different time stages of HRM. Each horizontal band represents a gene, and those with more than one homologous gene are ranked in Arabic order. Gene ID of this pathway all gene are listed Supplementary Table 6.

**Figure 4:**
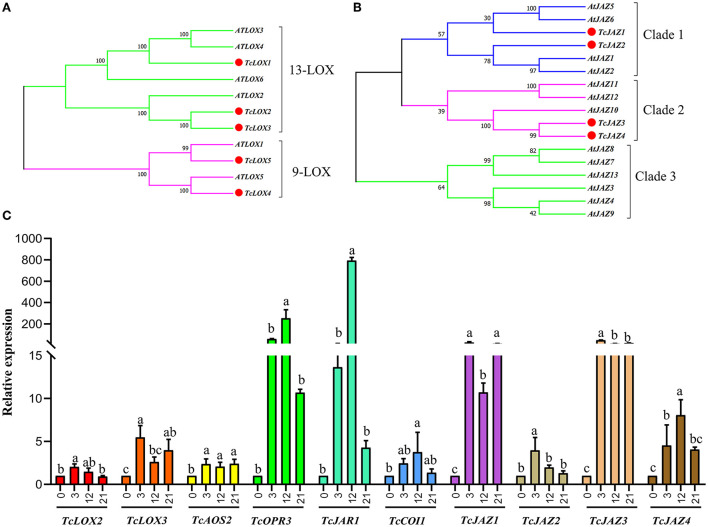
Phylogenetic trees of *T. ciliata LOXs* and *JAZs* and RT-qPCR analysis. The tree was constructed with the Maximum Likelihood method using MEGA program 7.0 with 500 bootstraps. **(A)** Phylogenetic tree of *LOXs* of *T. ciliata* and *Arabidopsis*. Five *LOXs* of *T. ciliata* named *TcLOX1-5* and are divided into two subfamilies, including 13-LOX and 9-LOX. **(B)** Phylogenetic tree of *JAZs* of *T. ciliata* and *Arabidopsis*. **(C)** Gene relative expression level of young stems on JA pathway. The English letters on the histogram are calculated according to Duncan's multiple range test, and different letters represent significant differences between the different groups at *P* ≤ 0.05.

Five *TcLOX* genes, two *TcAOS*, and one *TcAOC* genes were identified in the *T. ciliata* RNA-seq library. The expression levels of *TcLOX1/2/3/5* up-regulated 3–4 times when suffering 3 h HRM feeding, especially *TcLOX2* expression levels up-regulated continuously and up to maximum multiple (5) at 21 h (Figure 3). Phylogenetic analysis shows that *TcLOX1/2/3* belongs to 13-LOX subfamily and *TcLOX4/5* belongs to the 9-LOX subfamily. It suggests 13-LOX subfamily genes are the main force to resist HRM attacks in *T. ciliata* (Figure 4A). A *TcOPR3* gene was identified and expression level up-regulated 7-fold at 3 h (Figure 3). Although the expression level of *TcJAR1* increased at 3 h, it declined linearly in the subsequent period, indicating that its performance may be a stress response without the ability to resist insects (Figure 3).

In the RNA-seq library, four *TcJAZs* genes were identified and the expression level of *TcJAZ1/2/34* up-regulated by 13, 10, 73, and 20 times at 3 h, respectively. However, TcCOI1 expression level only changed slightly. The phylogenetic analysis of 13 *AtJAZ* and 4 *TcJAZs* genes *s*hows that *TcJAZ1*/2 belong to clade 1 and *TcJAZ3/4* belong to clade 2. Among them, *TcJAZ1* and *AtJAZ5/6, TcJAZ2* and *AtJAZ1/2*, and *TcJAZ3/4* and *AtJAZ10* are closely related, respectively, indicating these genes have similar function (Figure 4B). In addition, the expression levels of TFs regulated by JA, including ERF, WRKY, MYC, MYB, and NAC were significantly up-regulated. In conclusion, JA synthesis and signal transduction pathways were activated under HRM attack in *T. ciliata*. Q-PCR analysis shows that all tested genes were induced to express under HRM feeding, that is similar to RNA-seq data. Especially the relative expression levels of *TcOPR3, TcJAR1, TcJAZ1*, and *TcJAZ3* reached about 100 and it is worthy of further study (Figure 4C).

### Analysis of putative insect-resistant genes in terpene biosynthesis

**Figure 5:**
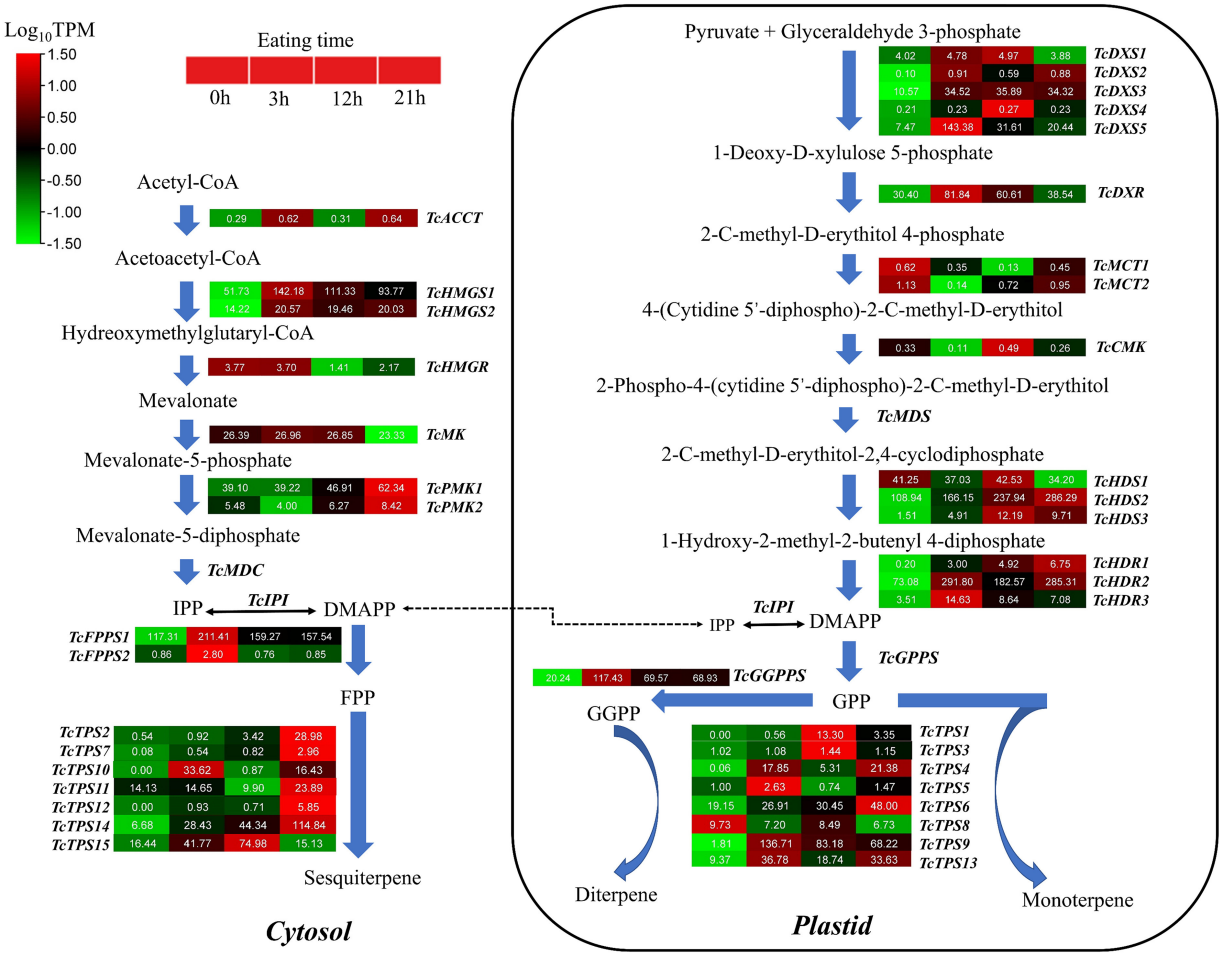
Gene expression patterns related to the biosynthesis of monoterpenes, sesquiterpenes, and triterpenes. The names of enzymes in each catalytic stage are represented by abbreviations and italics in bold. Color scales indicate gene expression levels (Log_10_TPM) at different time stages of HRM stress. Each horizontal band represents a gene, and those with more than one homologous gene are ranked in Arabic order. Gene ID of this pathway all gene are listed Supplementary Table 6.

After being gnawed by insects, plants will be induced to produce volatile compounds with anti-insect functions, such as terpenes. Therefore, we focused on digging out the terpene bio-related genes after HRM feeding of *T. ciliata*. In plants, sesquiterpenes are usually synthesized in the cytoplasm through the MVA pathway, which provides precursors for terpenoids in the cytoplasm or mitochondria. In the *T. ciliata* RNA-seq library, seven genes on the MVA pathway are identified, including a *TcACCT* gene, two *TcHMGS* genes, a *TcHMGR* gene, a *TcMK* gene and two *TcPMK* genes, but a *TcMDC* gene is not found (Figure 5). It is predicted that above genes don't contain signal peptides located in the cytoplasm (Figure 5). RNA-seq data shows that the expression levels of *TcACCT, TcHMGR*, and *TcMK* genes are low and don't change significantly. However, the expression levels of two *TcHMGS* and two *TcPMK* genes continue to up-regulate (Figure 5).

**Figure 6:**
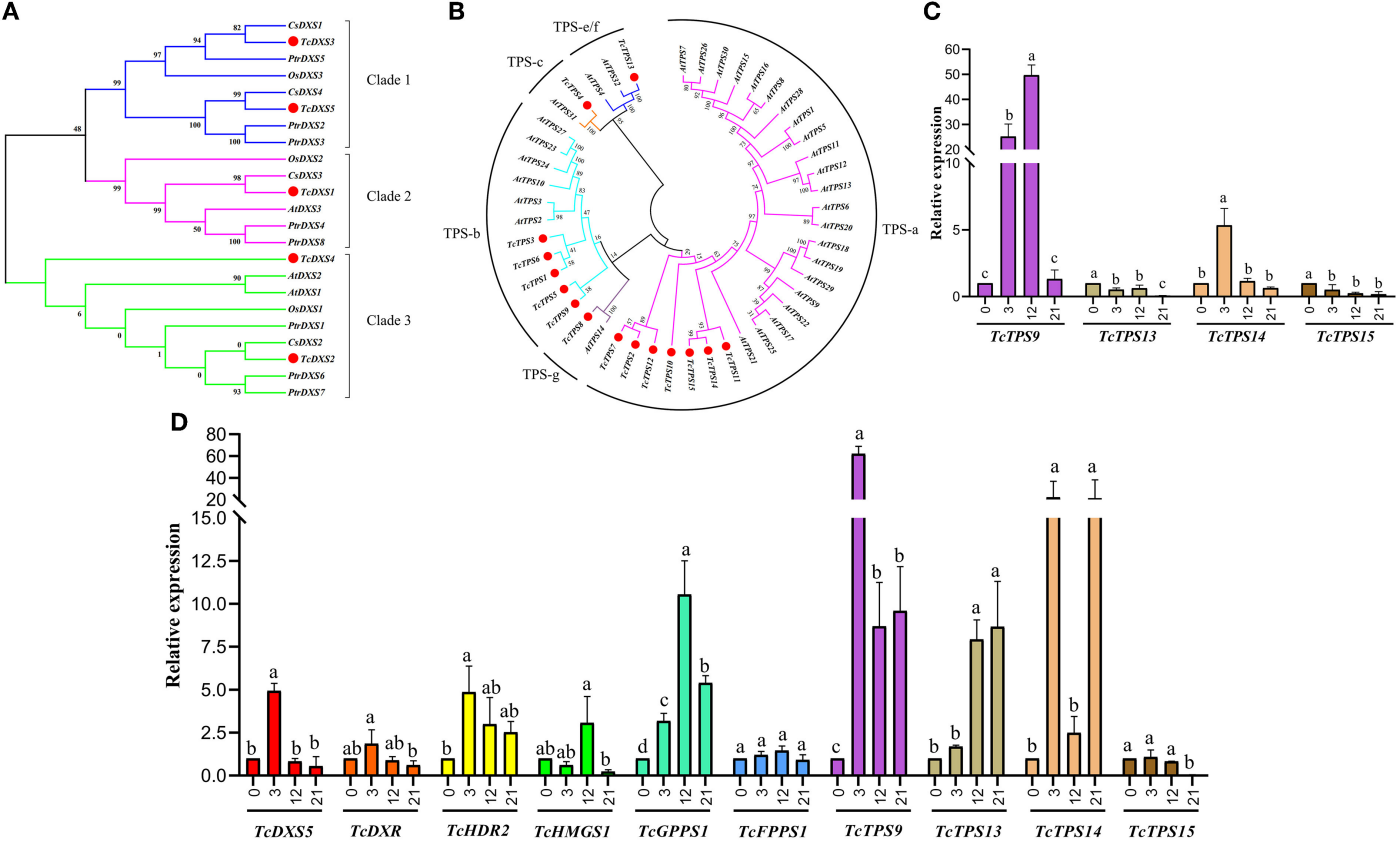
Phylogenetic trees of *T. ciliata DXSs* and *TPSs* and RT-qPCR analysis. The tree was constructed with the Maximum Likelihood method using MEGA program 7.0 with 500 bootstraps. **(A)** Phylogenetic tree of plant *DXSs*. Five *DXSs* of *T. ciliata* (named *TcDXS1-5*); *At, Arabidopsis; Cs, Citrus sinensis; Os, Oryza sativa; Ptr, Populus trichocarpa*. **(B)** Phylogenetic tree of *TPSs* of *T. ciliata* and *Arabidopsis*; All *TPSs* are divided into five subfamilies, including TPS-a, b, c, g, and e/f.: Gene relative expression level of leaves **(C)** and young stems **(D)** on MEP or MVA pathway. The English letters on the histogram are calculated according to Duncan's multiple range test, and different letters represent significant differences between the different groups at *P* ≤ 0.05.

By subcellular location prediction found above genes all located in plastids, which is consistent with the location of MEP pathway enzymes in *Arabidopsis* (Pokhilko et al., 2015). The first catalytic enzyme of the MEP pathway is *DXS*, which is also considered a rate-limiting enzyme. Studies have shown that the *DXS* gene family is generally divided into three different phylogenetic branches (Zhang et al., 2018). The expression of *DXS* genes in different evolutionary branches varies with development, tissue types, and environmental conditions. Phylogenetic analysis showed that *TcDXS3*/5 clustered to clade 1 is mainly used as a reference gene (Zhang et al., 2018; Figure 6A). Obviously, *TcDXS5* wasn't a reference gene but is involved in insect-resistance (Figure 5). Other genes expression level in the MVA pathway, such as *TcDXR, TcHDS*, and *TcHDR*, up-regulated at TC3 and TC12. However, the expression levels of *TcMCT* and *TcCMK* are significantly down-regulated, and there are large fluctuations, which may reflect the reduced demand of the main metabolism for metabolic flow (Yue et al., 2015).

The conversion between IPP and DMAPP is a reversible reaction. The reaction is catalyzed by isopentenyl pyrophosphate isomerase (*IPI*) to dynamically adjust the content of IPP and DMAPP (Lu et al., 2012). Then short-chain isopentenyl transferases (including *GPPS, FPPS*, and *GGPPS*) catalyze the head-to-tail condensation of IPP and DMAPP to isoprene GPP, FPP, and GGPP (Lu et al., 2012). There are two *FPPS* and one *GGPPS* in the *T. ciliata* RNA-seq library. The expression levels of the three genes all significantly up-regulated at TC3, TC12, and TC21 (Figure 5), indicating that *TcFPPSs* and *TcGGPPS* are involved in insect resistance.

Through KEEG annotation and homologous sequence search, 15 *TPS* genes were identified in the RNA-seq library. The subcellular location prediction shows that *TcTPS2/7/10/11/12/14/15* are located in the cytosol, and *TcTPS1/3/4/5/6/8/9/13* are located in the plastid (Figure 5). Among these genes, the *TcTPS4/6/9/13/14/15* gene's expression level significantly upregulated and requires further in-depth analysis. The *TPS* family in *Arabidopsis* is generally divided into five different subfamilies, including TPS-a, TPS-b, TPS-c, TPS-e/f, and TPS-g (Yu et al., 2020). Phylogenetic analysis showed that *TcTPS14/15* clustered into the TPS-a subfamily, which is mainly involved in monocotyledonous and dicotyledonous sesquiterpenes synthesis. *TcTPS6/9* clustered into the TPS-b subfamily, whose genes participated in angiosperm monoterpene biosynthesis. *TcTPS4* clustered into the TPS-c subfamily and *TcTPS13* clustered into the TPS-e/f subfamily (Figure 6B).

Q-PCR analysis showed that the expression level of *TcGPPS1, TcTPS9*, and *TcTPS14* was significantly up-regulated by about 10 times under HRM feeding (Figure 6D). Some studies have shown that TPS is a synthase of various terpenoids that have anti-insect effects (Schnee et al., 2006). Therefore, to further determine the functions of terpene synthase under HRM feeding, TcTPS*9/13/14/15* expression level in the leaves were detected. Interestingly, *TcTPS9* was significantly induced to up-regulate 60 times and gradually increase over time (Figure 6C), suggesting that *TcTPS9* catalyzes the synthesis of a certain terpenoid in the tender stem and leaf tissues under HRM feeding, but whether this substance is important for insect resistance to *T. ciliata* requires further study.

### Analysis of transcription factors responding to HRM

In order to discover the potential TFs with anti-insect function, the classification and expression level of TFs in the RNA-seq database were analyzed in depth. A total of 1,120 TFs are divided into 35 families (Supplementary Figure 3, Supplementary Table 7). The most abundant TF family is MYB (189), followed by NAC (121), AP2/ERF (120), and bHLH (86), C2C2 (72), WRKY (69), and bZIP (50). There are 323 (28.8%) differentially expressed TFs (DETFs) members, which are divided into 31 TFs families (Supplementary Figure 3). The MYB TF family contains the most DETFs, followed by AP2/ERF (36), C2C2 (35), NAC (32), bHLH (32), and WRKY (24) that were found in *Medicago truncatula* against aphid (Jacques et al., 2020). Then the detailed information and expression patterns of the top 40 TFs in terms of expression difference is listed on Table 1. Among the 9 MYB family members, only TRINITY_DN4447_c0_g1 is down-regulated under the HRM feeding, TRINITY_DN7191_c0_g1 and TRINITY_DN18799_c0_g1 are up-regulated by 36.8 and 20.35 times respectively at 3 h, which are sensitive to stress. But TRINITY_DN4331_c0_g1 and TRINITY_DN17485_c0_g1 are increased 19.36 and 13.88 times respectively at 21 h. It shows that the gene response time is inconsistent or there is functional redundancy between genes. In RNA-seq library of *T. ciliata*, there are 9 DETFs of AP2/ERF family, of which TRINITY_DN4586_c0_g1 and TRINITY_DN1335_c0_g2 are down-regulated and the other members were up-regulated of which TRINITY_DN8732_c1_g1 is up-regulated the most at 3 h (27.79 times). In addition, most members of the C2C2 TF family (5/7) are down-regulated, and TRINITY_DN14674_c1_g1, TRINITY_DN53864_c0_g1 are down-regulated by 44.52 and 33.92 times, respectively. Meanwhile, TRINITY_DN8705_c0_g1 is up-regulated by 21.73 times which may have potential to resist HRM. In addition, the bHLH (TRINITY_DN15909_c0_g1) is down-regulated by 24.77 times at 12 h. The genes mentioned above play a vital role in the process of *T. ciliata* resisting pests, and it is worthy of in-depth study in the later stage.

**Table 1:** Expression patterns of different TFs families with large differences in expression levels.

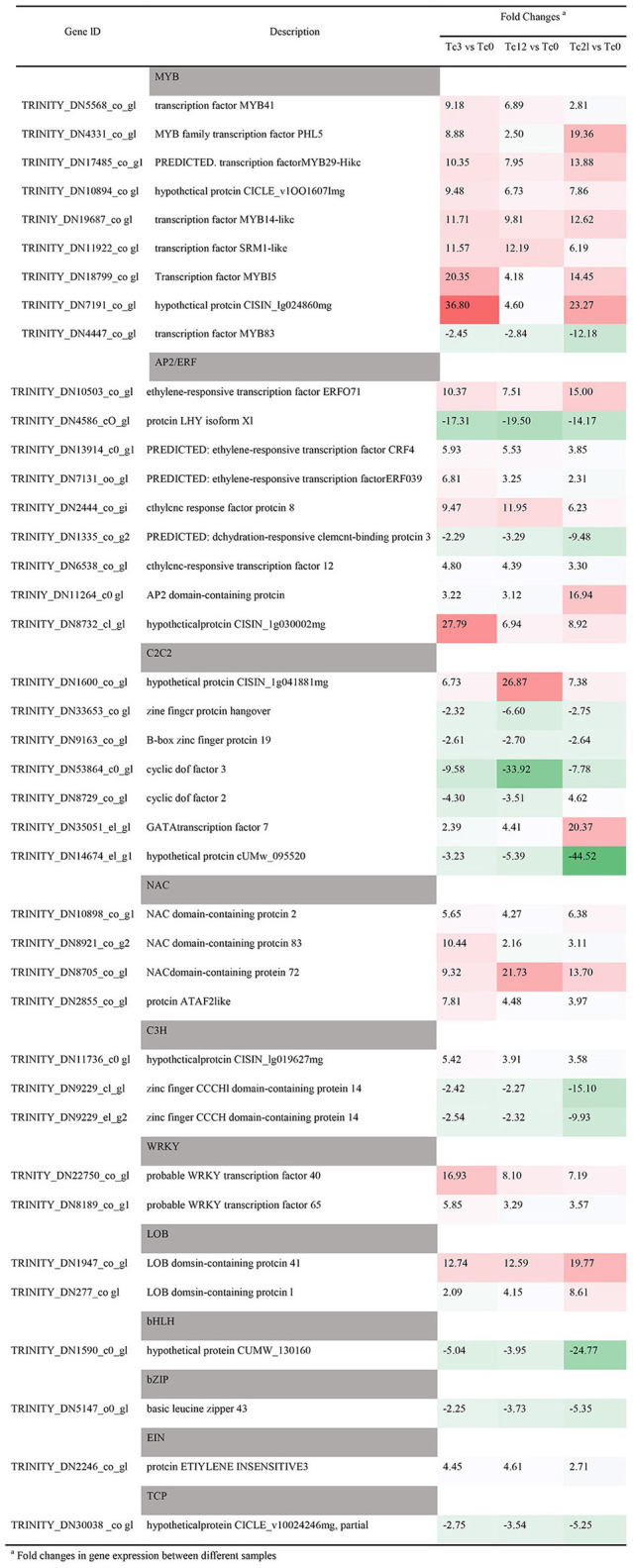

The green colour from dark to light indicates gene down-regulation fold from high to low and red colour from light to dark represents gene up-regulated fold from low to high.

### Gene expression validation

**Figure 7:**
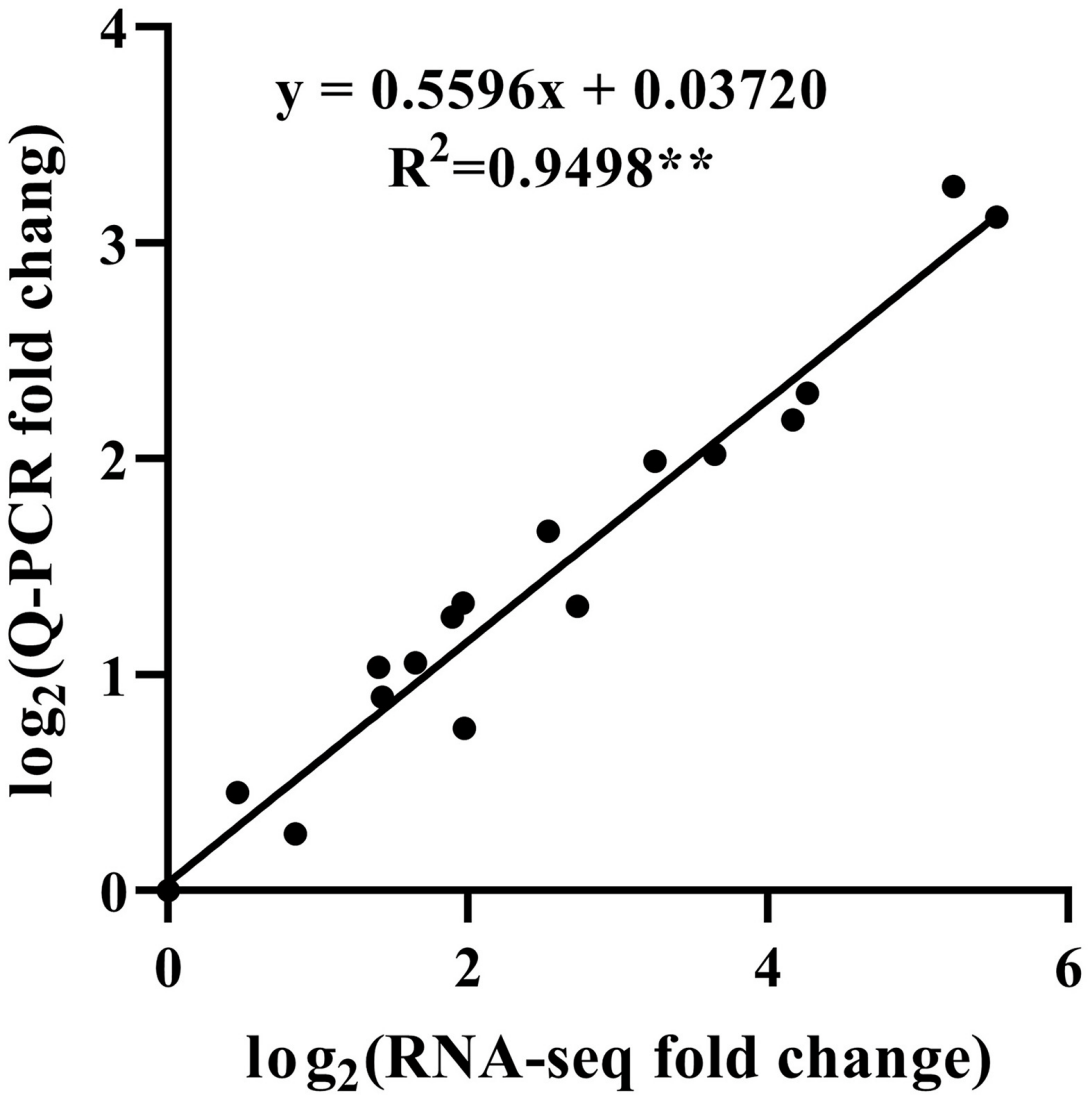
Correlation analysis of fold change obtained from Q-PCR and RNA-seq. Q-PCR fold change is the relative quantity of Tc3 (Tc12 or Tc21) normalized to expression level of Tc0, and RNA-seq fold change refers to the ratios of TPM values of Tc3 (Tc12 or Tc21) to Tc0 for selected transcripts. **Indicates a significant correlation at *P* < 0.001.

To validate the accuracy of the HRM-feeding *T. ciliata* transcriptome data, 20 single genes related to JA and terpene biosynthesis were selected for Q-PCR analysis (Figures 4C, 6D). The results showed that the gene expression profiles measured by Q-PCR and by DGE analysis were basically consistent. Linear regression analysis showed that the fold-change values of Q-PCR and RNA-Seq showed a significant correlation (*R*^2^ = 0.9498) at the level of *P* ≤ 0.001 (Figure 7). These results demonstrate the credibility of the RNA-Seq data generated in this study.

### Analysis of key metabolites in the JA pathway

**Figure 8:**
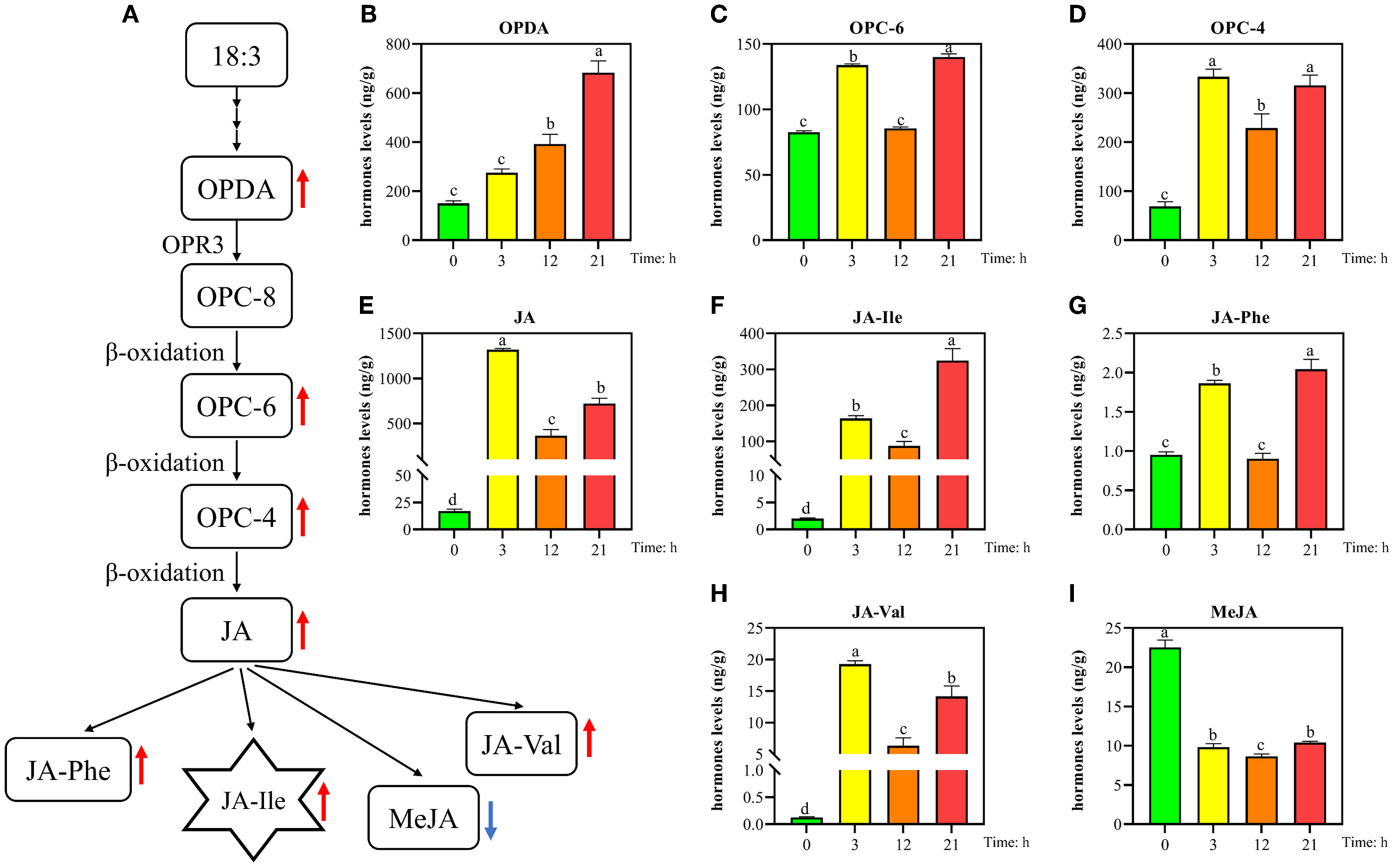
Simple diagram of JA synthesis pathway **(A)** and the variation of various metabolites at different times of HRM feeding **(B–I)**. 18: 3 for α-linolenic, OPDA for 12-oxo-phytodienoic acid, OPC-8 for 8-[3-oxo-2-(pent-2-enyl) cyclopentyl] octanoic acid, OPC6 for 6-[3-oxo-2-(pent-2-enyl) cyclopentyl] hexanoic acid, OPC4 for 4-[3-oxo-2-(pent-2-enyl) cyclopentyl] butanoic acid, JA-Ile for jasmonic acid-isoleucine, JA-Phe for jasmonic acid-phenylalanine, JA-Val for jasmonic acid-valine, and MeJA for methyl jasmonate. Red up-arrows indicate that hormone levels are rising and blue up-arrows indicate that hormone levels are declining. English letters on the histogram are calculated according to Duncan's multiple range test, and different letters represent significant differences between the different groups at *P* ≤ 0.05.

Transcriptome analysis found that JA synthesis and signaling pathways were significantly activated, and it also indirectly activated the MEP and MVA pathways. Therefore, in order to advance to confirm the important role of JA under HRM stress, the metabolites on the JA pathway were detected, including OPDA, OPC-6, OPC-4, JA, JA-Ile, JA-Phe, JA-Val, and MeJA (Figure 8). OPDA, OPC-6, and OPC-4 are precursors for the synthesis of JA, and their concentration levels were significantly increased (Figures 8A–D). The level of OPDA increased with the duration of HRM feeding. The level of JA in the untreated young stems of *T. ciliata* was 25 ng/g, reaching a maximum level (1,400 ng/g) at 3 h of HRM feeding, and remained at 500 ng/g at 12 and 21 h (Figure 8E). It shows that HRM feeding significantly induces the tender stems of *T. ciliata* to rapidly synthesize a large amount of JA. In addition, the concentration level of JA-Phe did not change (Figure 8G), MeJA decreased significantly (Figure 8I), and JA-Ile and JA-Val increased significantly (Figures 8F,H). Therefore, we speculate JA was mainly used as a substrate to synthesize JA-Ile and JA-Val under HRM feeding. Especially JA-Ile, its concentration at 3, 12, and 21 h was 100–300 times higher than untreated young stems (Figure 8F). JA-Ile binds to JAZ protein to activate downstream TFs to exert anti-insect function. At the same time, JA and JA-Ile can also indirectly activate the MVA and MEP pathways, which promote the production of terpenoids in plants to achieve self-defense.

## Discussion

In this study, the second instar larvae with a body length of 4–7 cm were selected for 12 h starvation culture which contributes to the smooth running of the gnawing experiment (Chen and Cha, 1998). And the larvae were placed on the young stems for gnawing at 9:00 in the morning, and all the larvae began to nibble within 20 min. Studies have shown that the HRM larvae can eat day and night, so the feeding time of the HRM will not affect the feeding effect (Chen and Cha, 1998). Some researchers choose to feed for a specific time and then detect the expression level at different times. However, the samples collected in our study are based on HRM feeding time that is based on two considerations. First, more serious damage degree will deeply and continuously stimulate defense mechanism. Second, HRM bores into the stem to feed, and there are a lot of yellow colloidal secretions like teardrops at the borehole which embed the HRM in the stem. Therefore, removal of the HRM at a specific time can cause more severe artificial mechanical damage, which can cause unnecessary transcription error (Lin et al., 2021). In *Arabidopsis thaliana, MYB3* has been shown to be involved in the synthesis of lignin and anthocyanin, regulating phenylpropane metabolism and other pathways, but no research has mentioned that it regulates the circadian rhythm, so it is preliminarily speculated that *MYB3* expression level is not affected by the time of day (Zhou et al., 2017; Kim et al., 2022). Moreover, The *MYB3* gene was selected as the reference of harvesting samples. First, previous studies have shown that *MYB* is more sensitive, which is also a very important marker (Zhai et al., 2017; He et al., 2020b; Jacques et al., 2020). Our results also showed that the greatest changes were at this stage and the trend of important genes was consistent, proving that this selection was reliable. Second, it is as unpredictable which key metabolites and which key genes will change when *T. ciliata* young stems are eaten by HRM. In this case, transcript detection is simpler and easier to perform than metabolite detection. In addition, according to the relative expression level of *TcMYB3*, the first sampling time (the time when the expression level of *TcMYB3* just started to be up-regulated) and the second sampling time (the expression level of *TcMYB3* reaching the maximum value) were first determined. The determination of the third sampling time is based on the time rhythm. That is to say, the interval between the third sampling time and the second sampling time is the same as the interval between the second sampling time point and the first sampling time point, so as to minimize the influence of time rhythm on transcripts.

**Figure 9:**
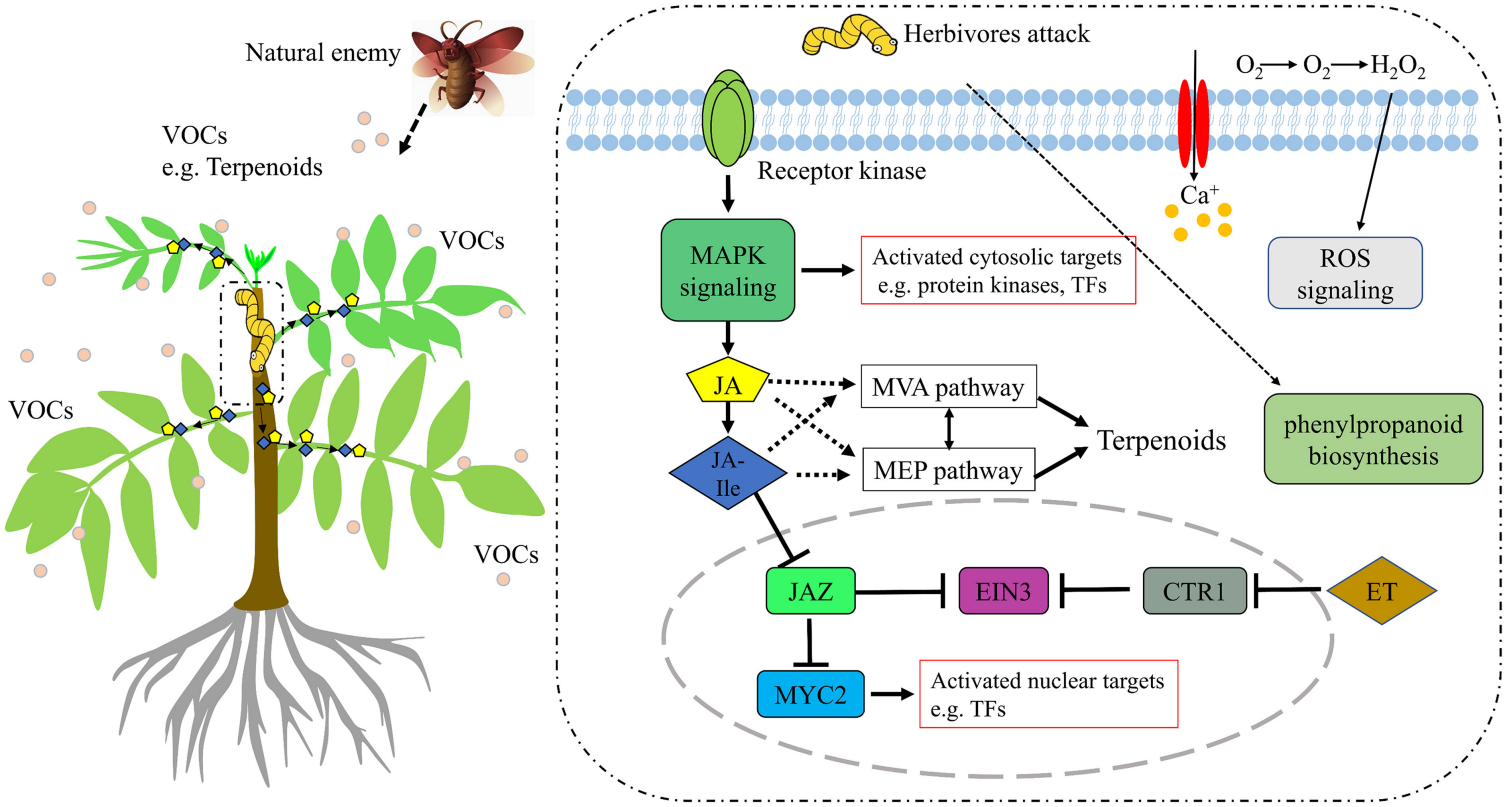
A hypothetical model of *T. ciliata* in repones to HRM. The response pathways include MAPK signaling, Plant hormone signaling (JA, ET), ROS signaling, and phenylpropanoid biosynthesis. MAPK, mitogen-activated protein kinases; ET, ethylene; EIN3, ethylene insensitive 3; CTR1, constitutive triple response 1; ROS, Reactive Oxygen Species. The dark blue diamond represents JA-Ile and the yellow pentagon represents JA.

Wounding damage from insect herbivores quickly triggers plant defense signals (Chen and Mao, 2020). The first reported damage-related peptide signal is system in that promotes the accumulation of JA and activates the expression of genes encoding protease inhibitors with insecticidal activity (Chen and Mao, 2020). In addition, other wound-inducing peptides have been found in plants, including *Arabidopsis* (Huffaker et al., 2006), rice (Shinya et al., 2018), and *maize* (Huffaker et al., 2011). Through transcriptome data analysis, JA synthesis-related genes, such as *LOX, AOS*, and *OPR3*, were significantly induced, which demonstrated JA accumulation (Figure 3). However, no wound-induced polypeptides were identified in *T. ciliata*. A Ca^2+^-binding protein kinase (CDPK) signaling pathway involved in crosstalk with mitogen-activated protein kinases (MAPKs) also leads to the formation of JA (Fürstenberg-Hägg et al., 2013). Meanwhile, in our RNA-seq library, MAPK signing pathway was significantly enriched (Figure 2E), and these pathway genes were also significantly poorly up- or down-regulated (Table 2). Therefore, when T. *ciliata* is eaten by HRM, calcium ion homeostasis is broken first, which acts as a second messenger in various plant signaling pathways. Then Ca^2+^ bind to protein kinases to activate MAPK signaling, thereby activating JA synthesis and signaling pathways (Figure 9). α-Linolenic acid (C18: 3) is the precursor of *trans*-jasmonic acid and *trans*-jasmonic acid is stress-related plant hormone that participates in the defense of insect herbivory and necrotizing pathogens (Killiny and Nehela, 2017). Therefore, it is speculated that α-linolenic acid, participates in the attack of HRM through induction of JA-mediated pathways, that exists in *citrus senensis* response to “*Candidatus* Liberibacter asiaticus” (Killiny and Nehela, 2017). *LOX, AOS*, and *OPR3* are the key genes supporting JA biosynthesis and are well-studied in other species. Studies show that feeding by *Spodoptera exigua* larvae on *Zea mays* induces expression of 9-lipoxygenases to a greater extent than 13-lipoxygenases (Woldemariam et al., 2018). However, in our study, *Tc-LOX1/2/3* belonging to 13-LOX subfamily are the main force to resist HRM attacks in *T. ciliata* (Figures 3, 4A), which provides a new perspective on insect resistance research. *OsAOS1* and *OsAOS2* had been proved to play a vital role in determining the resistance of rice to chewing and phloem-feeding herbivores (Zeng et al., 2021). Similar to *OsAOS1* and *OsAOS2, TcAOS2* expression level also up-regulated 8-fold at 3 h, suggesting *TcAOS2* has great potential to resist HRM attack. In other plants, such as *Arabidopsi*s (Body et al., 2019), tomato (Bosch et al., 2014), and potato (Schoenherr et al., 2019), OPR3 all have the function of resisting pests, which indicates that *TcOPR3* is worthy of further study. The increasing concentrations of metabolites, OPDA, OPC-8, OPC-6, and OPC-4, under HMR stress indicated that *TcOPR3* plays an irreplaceable role as an upstream gene of JA synthesis (Figure 8). In *Arabidopsis*, OPR3 acts as a branch point between high susceptibility and wild-type-like disease levels, suggesting a role for OPDA in regulating plant defenses against root-knot nematodes (*Meloidogyne hapla*; Gleason et al., 2016). JA is converted to different active and inactive compounds during the plant stress response and development, including 12-OH-JA, MeJA, JA-Ile, JA-Val, and JA-Phe (Wasternack and Song, 2017). Metabolic analysis showed that only the concentration levels of JA and JA-Ile produced huge increases after HRM feeding (Figure 8), suggesting that JA is mainly catalyzed to synthesize JA-Ile which is most biologically active JA compound (Wasternack and Song, 2017). JA-Ile will be transported to the nucleus to negatively regulate JAZ, then JAZ inhibits MYC2 while negatively regulating EIN3 and ET. Downstream transcription factors will be activated to participate in insect resistance, such as MYB, ERF, NAC, etc. An amount of TFs from different TFs families have been shown to play a key role in regulating the pest stress in *Arabidopsis*, rice, wheat, and many other plant species (Naidoo et al., 2014). For example, *TaMYB19, TaMYB29*, and *TaMYB44* are co-regulators against aphid of wheat (Zhai et al., 2017) and *AtERF5, AtERF6*, and *AtRAP2.2* have been implicated in resistance to Botrytis cinerea mediated by JA and ethylene in *Arabidopsis* (He et al., 2020a).

**Table 2:** Expression patterns of other pathway under HRM stress, include MAPK signaling, Phenylpropanoid biosynthesis, and Plant hormone signal transduction.

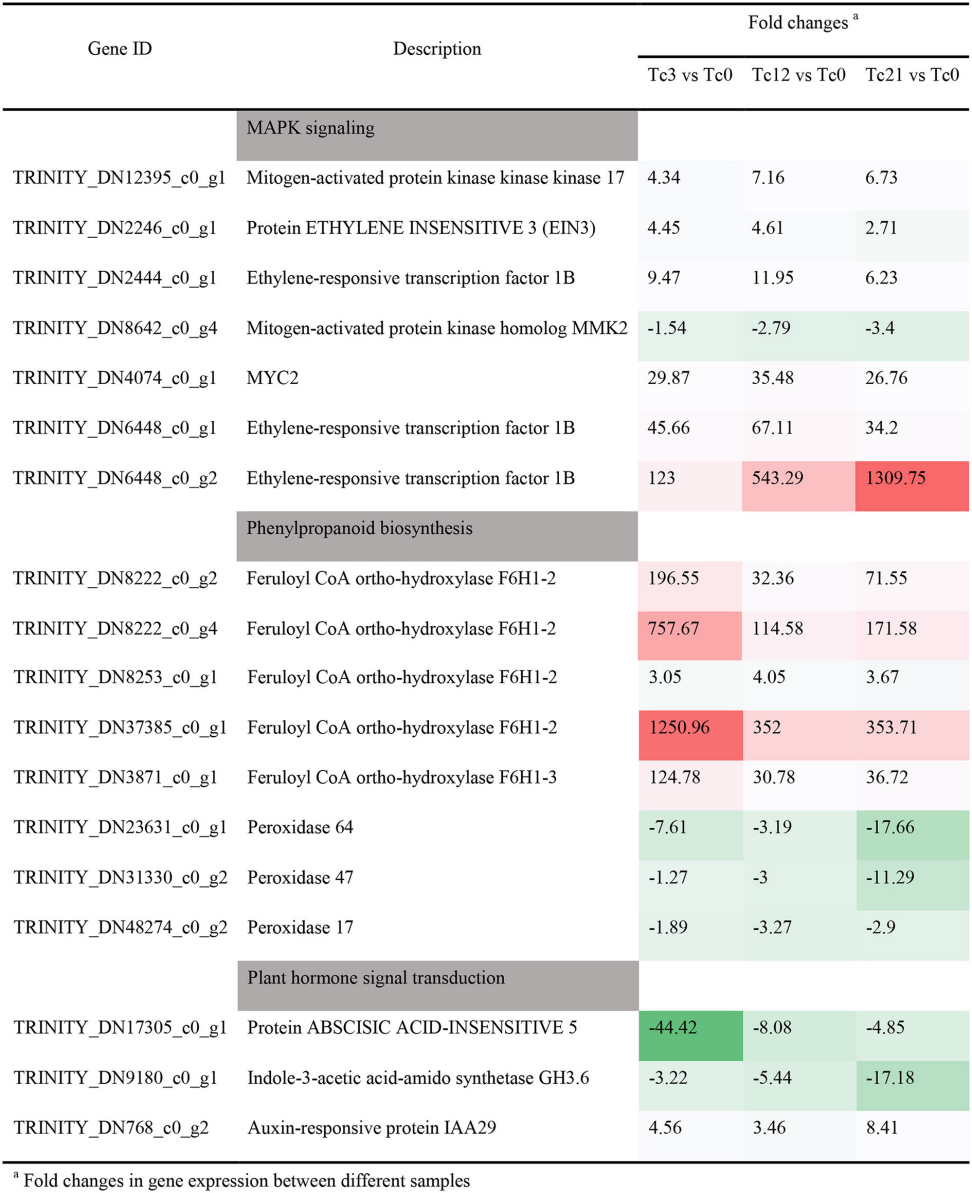

The green colour from dark to light indicates gene down-regulation fold from high to low and red colour from light to dark represents gene up-regulated fold from low to high.

In this study, most DEGs are mainly enriched in oxidoreductase activity, dioxygenase activity, protein disulfide oxidoreductase activity, etc., which suggests *T. ciliata* may activate xanthine oxidoreductase through MAP kinase-dependent pathways (Abdulnour et al., 2006) in order to protect itself. Meanwhile, the rhythm-related pathways are detected due to the difference in sampling time, such as entrainment of circadian clock, positive regulation of circadian rhythm, circadian rhythm, etc. (Gil and Park, 2019). Also, there are 323 TFs from 31 families were identified, which responded HRM feeding (Supplementary Figure 3). Among them, MYB, AP2/ERF, C2C2, and NAC families comprise a high proportion of pest stress-responsive members. NAC (NAM, ATAF1/2, and CUC) domain proteins play a role in diverse processes, including development, morphogenesis, senescence, and stress responses and studies show that *ATAF1* and *ATAF2* genes were highly induced by wounding and pathogen infection (Ooka et al., 2003; Delessert et al., 2005; Wang et al., 2009). Meanwhile, *IbNAC1*, upregulates sporamin gene expression by binding the SWRE motif against mechanical wounding and herbivore attack in potato (Chen et al., 2016). On the other hand, JA-Ile transported to other cells or tissues affects plant volatile organic compounds (VOCs) emissions and JA-mediated changes in monoterpenes may protect trees under biotic and abiotic stress (Filella et al., 2006; Mujiono et al., 2020). Spraying MeJA on Norwegian spruce (*Picea abies*) can induce the accumulation of terpenoids in its resin canal to effectively deal with the damage of beetles (Mageroy et al., 2020). In *Arabidopsis*, the ability of plants to produce JA determines the emission of some herbivorous-induced volatiles such as terpenoids and methyl salicylate, but not green leaf volatiles (GLV; Snoeren et a., 2009). In *Nicotiana attenuata*, JA is a more potent stimulator of (E)-α-bergamot emission than JA-Ile, suggesting that JA-Ile modulates specific aspects of herbivore resistance. This specificity may allow plants flexibility in the trade-off between herbivore resistance and growth and reproduction during ontogeny (Schuman et al., 2018). Therefore, the dramatic increase in JA and JA-Ile under HRM stress may means that both are involved in regulating the MEP and MVA pathways in *T. ciliata* (Figures 8, 9). In plants, there are over 1,000 volatile organic compounds (VOCs), mainly composed of 6-carbon aldehydes, alcohols, esters and various terpenes. VOCs are used to attract pollinators and predators or repel herbivores as well as in communication between or within plants (Fürstenberg-Hägg et al., 2013). The synthetic pathways of terpenoids, MVA, and MEP, in the young stems of *T. ciliata* were activated, and *TcTPS9* expressed highly in leaf tissues. Therefore, it is inferred that JA and JA-Ile are transported into leaf cells to activate key insect resistance genes' expression that can synthesize signal substances to attract natural enemies or communicate with peers (Figure 9). Rice, fed on by fall armyworms, will induce volatiles and the corresponding volatile biosynthetic genes potentially involved in indirect defense (Yuan et al., 2008). HMGR is a synthesis rate-limiting enzyme of the MVA pathway. In this study, HMGRs were activated to promote the synthesis of terpenoids (Figure 5). Also, *HMGS* and *HMGR* were highly expressed in tomato, which produce many secondary metabolites that act against pathogens and pests (Liao et al., 2014). Moreover, phenylpropanes derivatives, such as flavonoids, coumarins, lignin, and phenolamides, will rapidly accumulate to higher levels as components of an induced defense arsenal against herbivore attack in wild tobacco *Nicotiana attenuata* (Gaquerel et al., 2014). At the same time, biosynthesis pathway genes were induced expression significantly at TC3 (Table 2), especially TRINITY_DN8222_c0_g4 (757-folds) and TRINITY_DN37385_c0_g1 (1,250-folds), this suggests phenylpropane biosynthesis pathways may participate in the insect-resistance mechanism by producing phenylpropane derivatives in *T. ciliata*, which needs further experiments to prove.

## Conclusion

In this study, a comparison of transcriptomic profiles from young *T. ciliata* stems subjected to 0, 3, 12, and 21 h HRM feeding was performed using RNA-Seq. A total of 84,227 unigenes were generated from 12 samples, and 41,568 unigenes were annotated in different databases. Many DEGs were identified that were mainly involved in JA synthesis, signal transduction, and terpene biosynthesis. At the same time, many key genes have been found in these biological pathways, including *TcOPR3, TcJAR1, TcJAZs*, and *TcTPS9*. These genes are worthy of follow-up in-depth study. This study not only provides insights into the molecular mechanisms underlying the resistance of *T. ciliata* to HRM but also helps to explore new biocontrol strategies against insects in eco-friendly woody plants.

## Data availability statement

The data presented in the study are deposited in the SRA repository in submission SUB11223109 (https://submit.ncbi.nlm.nih.gov/subs/bioproject/SUB11223109/overview), accession number are SRR18680895, SRR18680894, SRR18680893, SRR18680892, SRR18680891, SRR18680890, SRR18680889, SRR18680888, SRR18680887, SRR18680886, SRR18680885, and SRR18680884.

## Author contributions

PL and HS conceived and designed research. HS performed the experiments and wrote the manuscript. HS, YL, ZW, and ZD analyzed the data. YW and EY performed the RT-qPCR experiment. QQ, PL, and XC guided the experiment and revised the manuscript. All authors read and approved the final manuscript.

## Funding

This work was supported by National Key R&D Program of China (Grant No. 2021YFD2200305), Guangdong Basic and Applied Basic Research Foundation (Grant No. 2021A1515010534), Science and Technology Project of Guangzhou (Grant No. 202102080217), and Characteristic Innovation Projects of Department of Education of Guangdong Province (Grant No. 2019KTSCX017).

## Conflict of interest

The authors declare that the research was conducted in the absence of any commercial or financial relationships that could be construed as a potential conflict of interest.

## Publisher's note

All claims expressed in this article are solely those of the authors and do not necessarily represent those of their affiliated organizations, or those of the publisher, the editors and the reviewers. Any product that may be evaluated in this article, or claim that may be made by its manufacturer, is not guaranteed or endorsed by the publisher.
