# Supplementary figures and images for: Transcriptome profiling of Toona ciliata young stems in response to Hypsipyla robusta Moore
Source: Front Plant Sci. 2022 Aug 25;13:950945. doi: 10.3389/fpls.2022.950945 (PMC9465623; doi:10.3389/fpls.2022.950945)

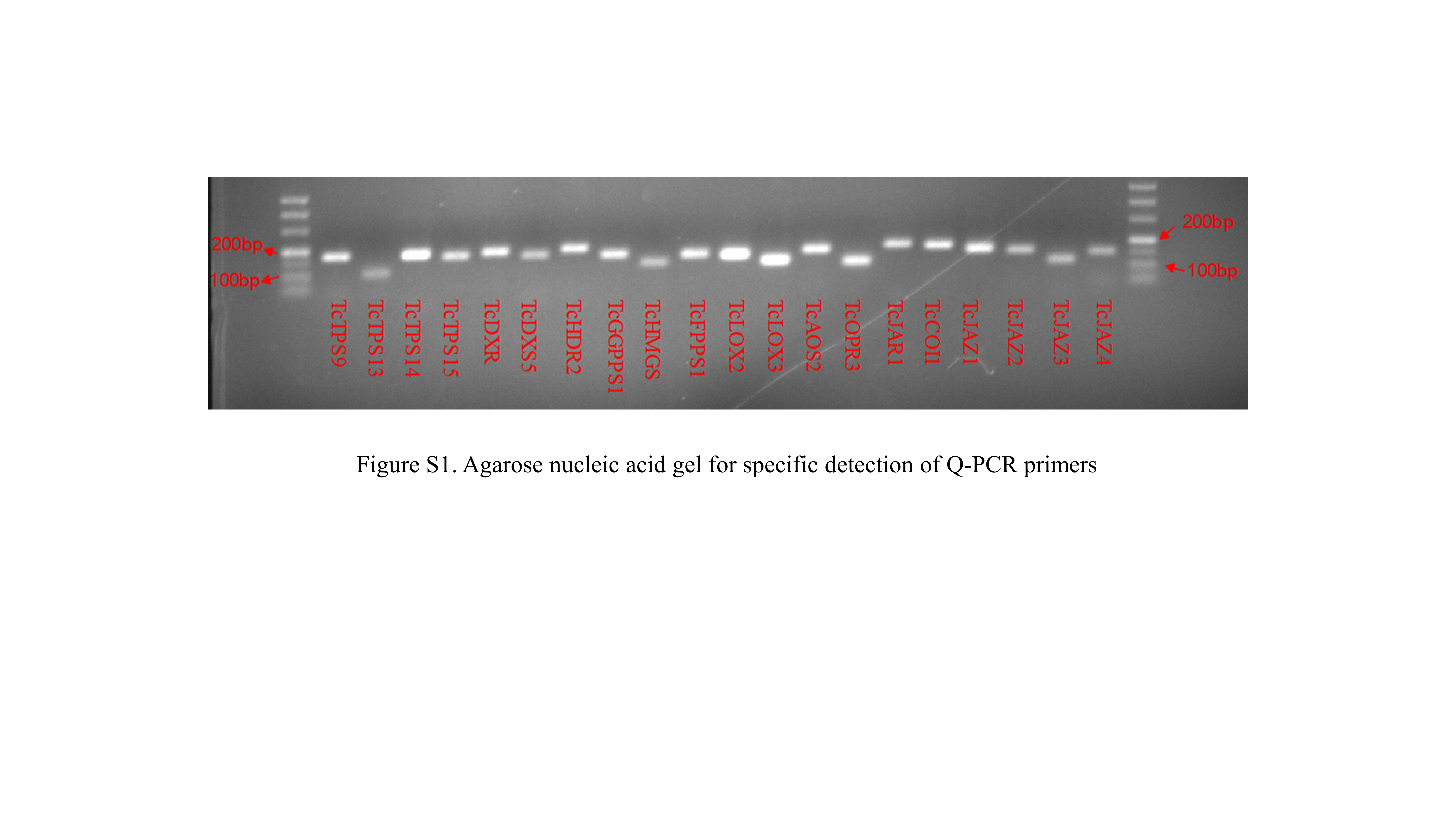

Supplement: Supplementary file 1 [file Image_1.tif]

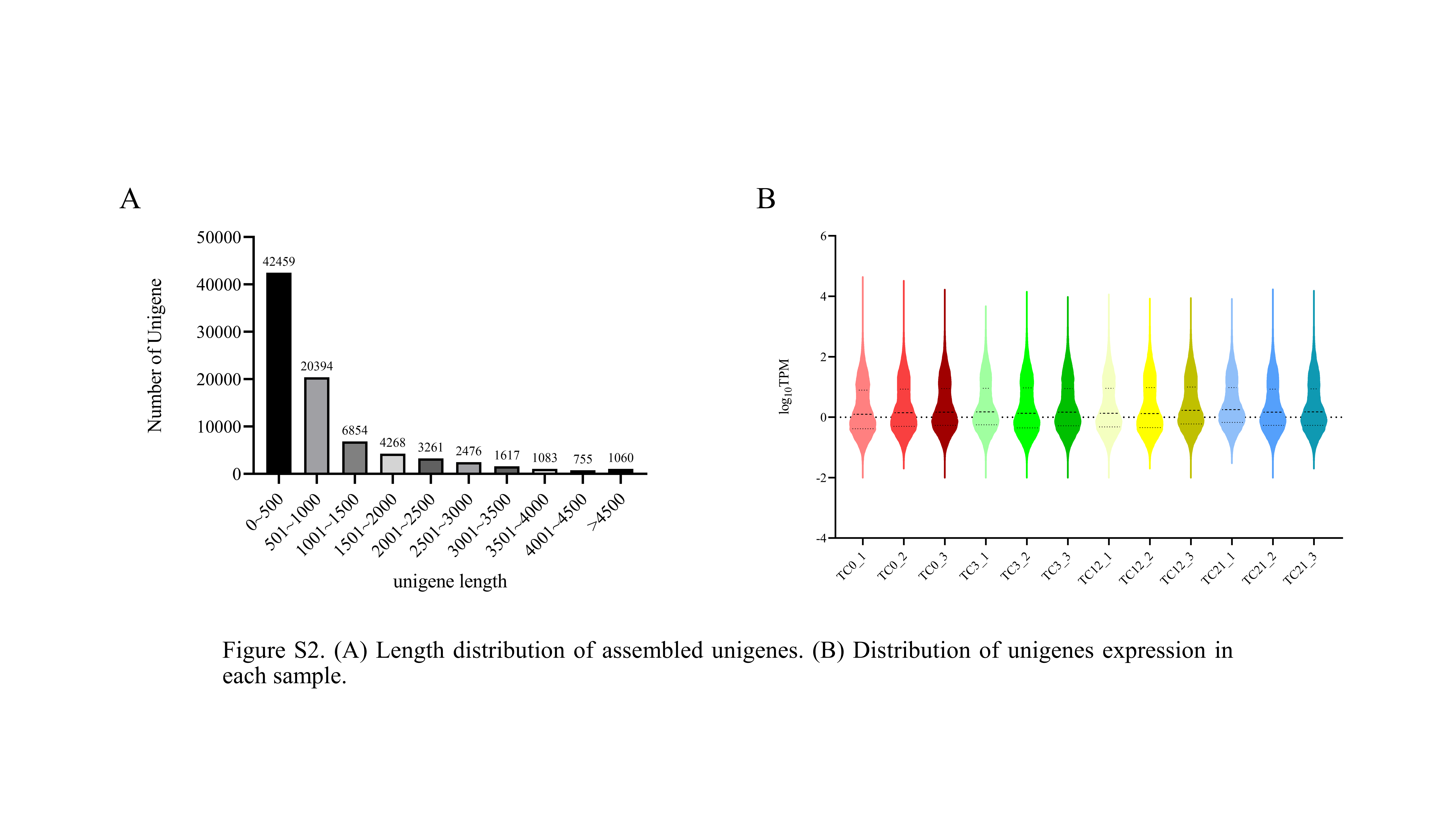

Supplement: Supplementary file 2 [file Image_2.tif]

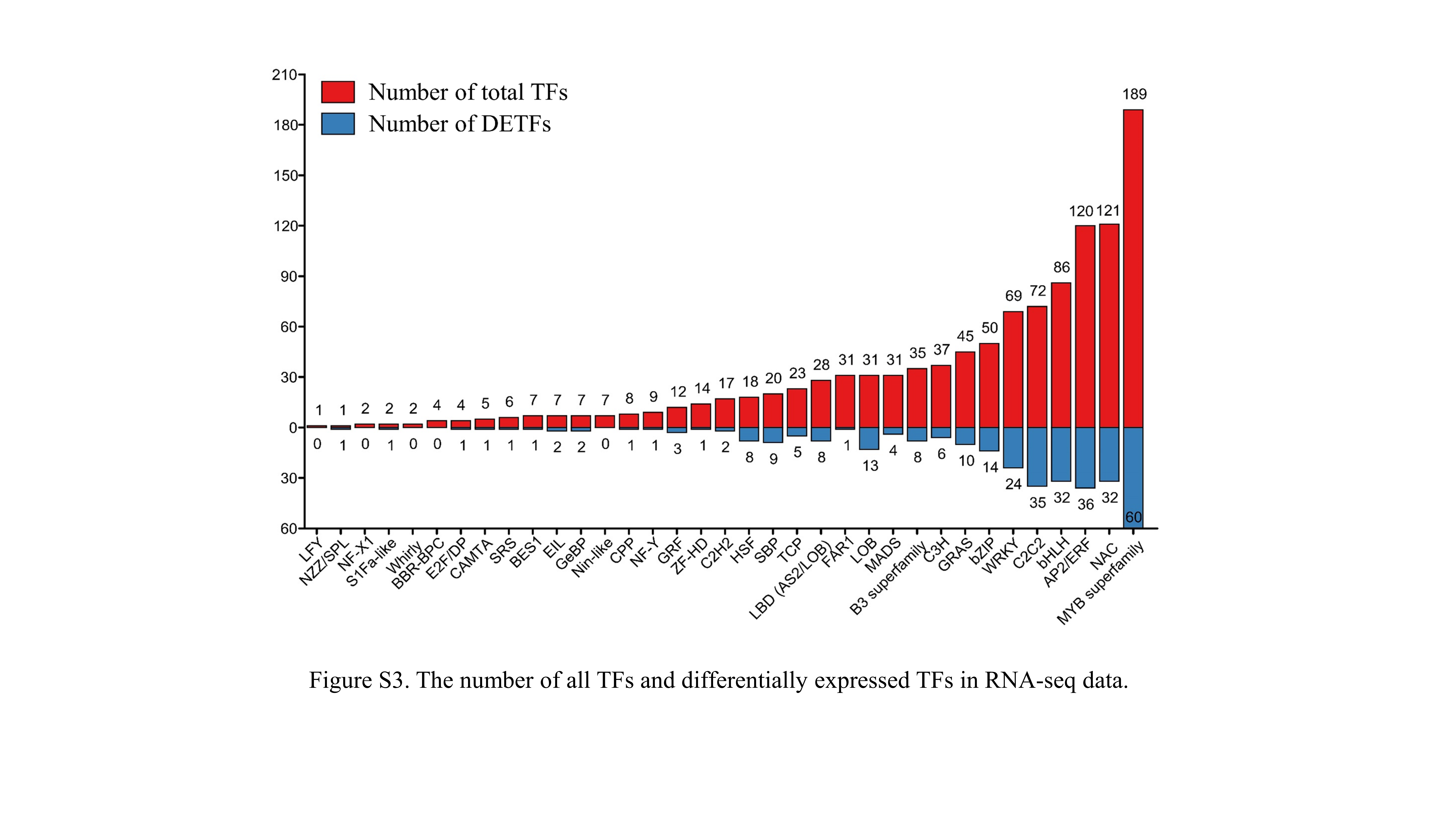

Supplement: Supplementary file 3 [file Image_3.tif]
